# *Galleria mellonella* (greater wax moth) larvae as a model for antibiotic susceptibility testing and acute toxicity trials

**DOI:** 10.1186/s13104-017-2757-8

**Published:** 2017-08-29

**Authors:** Katarzyna Ignasiak, Anthony Maxwell

**Affiliations:** Department Biological Chemistry, John Innes Centre, Norwich Research Park, Norwich, NR4 7UH UK

**Keywords:** *Galleria mellonella*, Infectivity testing, Toxicity trials, Antibiotics

## Abstract

**Background:**

Infectivity trials and toxicity testing in rodents are important prerequisites to the use of compounds in man. However, trials in rats and mice are expensive and there are ethical considerations. *Galleria mellonella* (greater wax moth) larvae are a potential alternative. We have assessed the use of these insects in infectivity trials and toxicity testing.

**Findings:**

Using four bacterial species (two Gram-negative and two Gram-positive) we have assessed the efficacy of four antibiotics against infections in Galleria and compared the antibiotic susceptibility with that in humans. In general, we find a good correlation. Similarly, we have assessed 11 compounds (initially tested blind) for their toxicity in Galleria and compared this with toxicity trials in mice and rats. Again we found a good correlation between toxicity in Galleria and that in rodents.

**Conclusion:**

We have found, in our hands, that *G. mellonella* larvae can be used in infectivity trials and toxicity testing, and that these assays represent an inexpensive and readily executable alternative to testing in rodents.

## Background

It is of vital importance that compounds intended for use in humans are adequately tested in suitable animal systems. Rodents (rats and mice) are commonly used in this regard, but cost and ethics have to be carefully considered. For example, the 3Rs (Replacement, Reduction and Refinement) are increasingly seen as a framework for conducting high quality science in the academic and industrial sectors with more focus on developing alternative approaches that avoid the use of animals [1]. It is therefore useful to contemplate alternative invertebrate models, if these can be shown to yield useful data. The greater wax moth (*Galleria mellonella*) is an insect in the order Lepidoptera and its larvae have previously been used for virulence and antimicrobial efficacy studies [2]. There are many benefits to using wax-moth larvae. Many larvae can be used in each experiment making pharmacokinetic and pharmacodynamic data easy to obtain. Pharmacokinetic data obtained in *G. mellonella*, such as antibiotic clearance time, elimination half-time of the drug and maximum drug concentration, can directly correlate to human data [3]. Numerous studies confirm that microbial pathogenicity and virulence determinants are the same in humans, mice and wax moths [4–6]. The insects can be bred quickly (at 37 °C the full life cycle lasts ~6 weeks) at low cost and without the need for specialized equipment; in addition they are not generally subject to ethical considerations. Galleria larvae are large, reaching 250–300 mm in length at fifth instar, enough for intraperitoneal injection of test compounds. Additionally, the insect immune system is functionally and structurally similar to the mammalian innate immune system [7, 8].

*Galleria mellonella* larvae were first used to assess antibiotic efficacy against *Acinetobacter baumannii* [9]. Cefotaxime, tetracycline, gentamicin and meropenem were assayed against a systemic *A. baumannii* infection. Gentamicin and meropenem, which *A. baumannii* is susceptible to, significantly prolonged the survival of infected larvae, while the survival of infected but untreated larvae and larvae treated with cefotaxime and tetracycline, which the bacteria are resistant to, was less than 25% in 5 days. The model quickly gained interest with a number of academic groups using it as a standard testing model. *G. mellonella* larvae have been used to investigate emerging pathogens [10] and novel treatments for persisting pathogens [3].

Toxicity testing in *G. mellonella* is an extrapolation from antibiotic efficacy studies. Antibiotic efficacy studies establish a dose of an antibiotic necessary to clear a bacterial infection. One of the necessary controls in antibiotic efficacy testing is to ensure that the antibiotic itself is not toxic to the insects. Apart from establishing a safe dose for efficacy testing a LD_50_ dose (median lethal dose; a dose of compound that is sufficient to kill 50% of a population of test animals) can be measured. However only recently have *G. mellonella* larvae been used in de novo toxicity testing. The toxicity of ionic liquids, which are low temperature molten salts used as an alternative to volatile solvents, has been assayed [11]. Ionic liquids are commonly labelled “ecologically friendly”, even though the class is diverse and exhibits a wide range of toxicities. In this study the systemic toxicity was correlated to the length of alkyl chains of the 1-alkyl-3-methylimidazolium ionic liquids tested. The salts were toxic under 100 µg/g, apart from the shortest two-carbon alkyl chain salt (1-ethyl-3-methylimidazolium chloride) which had a LD_50_ of nearly 8000 µg/g, presenting a negligible toxicity. Interestingly, even though their rising popularity as an alternative to volatile organic compounds, 1-alkyl-3-methylimidazolium ionic liquids lack toxicity data.

The aim of the present work was to evaluate the effectiveness of *G. mellonella* in antibiotic susceptibility trials and to explore whether they could also be routinely used in acute toxicity assays. Specifically, for the antibiotic efficacy trials we wanted to establish if the therapeutic doses established in the wax moth larvae correlate with doses recommended for human use, and for the toxicity testing we wanted to determine if the LD_50_ values established for wax moth larvae correlate to values established in rodents.

## Methods

### Bacterial strains and media

We chose two Gram-positive and two Gram-negative bacterial strains that are of clinical relevance for these studies; these were: *Escherichia coli* (ATCC 25922), *Mycobacterium smegmatis* (ATCC 700084), *Pseudomonas aeruginosa* (ATCC 15692) and *Staphylococcus aureus* (ATCC 29213), and were obtained from the Health Protection Agency culture collection (Public Health England, Porton Down, UK). They were cultured from glycerol stocks and maintained on appropriate media on agar plates [Middlebrook medium (BD Difco 7H9) for *M. smegmatis*, LB (LMM0202, Formedium) for all others] before growth in LB broth aerobically at 37 °C. Cultures were sub-cultured at least twice before being used in the assays.

### Compounds

Ampicillin, ciprofloxacin, rifampicin and tetracycline for antibiotic efficacy testing were obtained from Sigma Chemicals. For toxicity testing of compounds supplied by Inspiralis Ltd., sources were: ciprofloxacin (Fluka), etoposide, novobiocin, amsacrine, NaCl, tetracycline, DMSO, chloroquine, streptomycin and ATP (Sigma), chloramphenicol (Duchea Biochemie), doxorubicin (Calbiochem) and glucose (Fisher Chemicals) (Table 1). Due to insolubility issues, amsacrine was supplied at 8 mg/ml in 50% DMSO in water; doxurubicin was supplied at 5.5 mg/ml in 50% DMSO in water. For toxicity trials all compounds were initially provided in numbered tubes without compound names to avoid bias. The identity of the compounds was revealed only when the testing procedure was completed, and the data from *G. mellonella* was compared to material safety data sheet (MSDS) pages available with the compounds.

**Table 1:** Compounds used in this study

Compound	Stock concentration (mg/ml)	Notes
Ampicillin	25	Supplied in 50% DMSO in water
Amsacrine	8^a^	Supplied in 50% DMSO in water^a^
Chloroquine	25	Supplied in 10% DMSO in water
Ciprofloxacin	25	Supplied in 10% DMSO in water
DMSO	N/A	Negative control
Doxorubicin	5.5^a^	Supplied in 50% DMSO in water
Etoposide	25	Supplied in 50% DMSO in water
Glucose	25	Supplied in 10% DMSO in water
Novobiocin	25	Supplied in 50% DMSO in water
Rifampicin	25	Supplied in 50% DMSO in water
Sodium chloride	25	Supplied in 10% DMSO in water
Streptomycin	25	Supplied in 10% DMSO in water
Tetracycline	25	Supplied in 10% DMSO in water

*N/A* not applicable

^a^Lower concentration due to insolubility

### Insect rearing

A colony of *G. mellonella* was obtained from the John Innes Centre Entomology Facility (originally sourced from Livefood UK Ltd.). The colony was kept in the dark at 37 °C in large Petri dishes (140 mm, Sterilin) filled with artificial food. The artificial food was composed of 300 ml honey (Sainsbury’s Honey, Clear), 400 ml glycerol (G5516, Sigma Chemicals), 200 g milk powder (Dried Skimmed Milk Powder, Marvel), 200 g wholemeal flour (Strong Stoneground 100% Wholemeal Flour, Sainsbury’s), 100 g yeast powder (103753, Merck), 100 g wheat germ (Neal’s Yard Wholefoods Natural Wheatgerm) and 400 g bran (Neal’s Yard Wholefoods Natural Wheat Bran). First, the dry and wet ingredients were mixed separately, and then the mixtures were combined. The diet was mixed with beeswax pellets at a 2:1 ratio. Unused food was stored at 4 °C. The food was replaced at least once per week, unless not enough was left for the larvae to feed on, in which case more food was added to the containers.

### *G. mellonella* injection procedure

Five to ten larvae (250–320 mg each) were selected at random for each step in the procedure. Any larvae with darkening of the cuticle were discarded. The test compounds were injected into the hemocoel in DMSO or PBS buffer through the last left proleg (Hamilton syringe 701N, volume 10 μl, needle size 26 s, cone tip) [10] unless stated otherwise. The larvae were placed on medical tissues (Kimtech) to stop the hemolymph from leaking. The larvae were incubated in the dark for 5 days and mortality was recorded daily.

### Determination of the infective dose of bacteria

An infective dose of bacteria was determined by injecting groups of five larvae with bacterial suspension at: 5 × 10^4^ colony forming units (cfu) per injection, 5 × 10^5^ cfu, 5 × 10^6^ cfu and 5 × 10^7^ cfu. The larvae were incubated for 5 days. An infective dose was defined as one that caused an immune response, recognizable by the darkening of the cuticle [10]. In *G. mellonella* larvae immune response leads to the formation of melanin plaques around bacteria immobilized by the cells of the immune system. These plaques appear dark through the cuticle. An infective dose of bacteria was one that caused 60–80% lethality within 48 h, but not 100% lethality within 24 h. The larvae were incubated at 37 °C as bacterial virulence changes with temperature and the experiment was designed to mimic infection in humans.

### Antibiotic efficacy testing

**Fig. 1:**
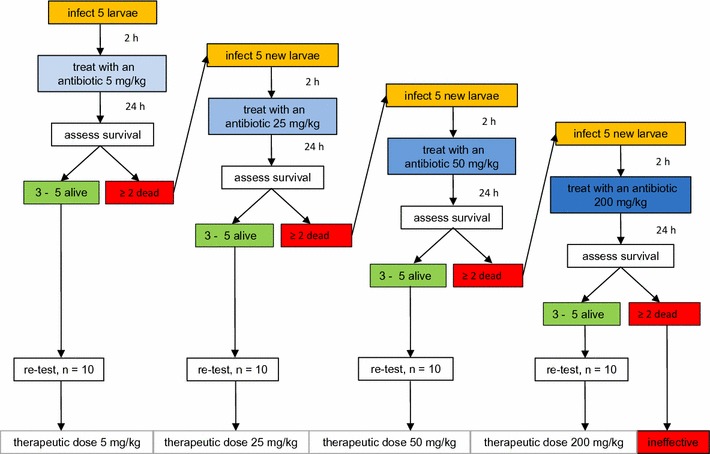
A flowchart representing consecutive steps in the antibiotic efficacy test. A starting dose of 5 mg/kg body weight was administered and the insects were scored for survival. If the mortality was under 40%, the compound was assigned the lowest therapeutic dose. If the mortality was over 40%, a higher dose was tested until a therapeutic dose was established

A flowchart was used to assign an antibiotic therapeutic dose against a panel of bacteria (Fig. 1). For the antibiotic efficacy experiment five larvae (as recommended by OECD guidelines) were injected into the last left proleg with a pre-determined infective dose of bacteria and incubated for 2 h at 37 °C [3, 12, 13]. After the incubation the larvae were injected in the last right proleg with the lowest dose of antibiotic (5 mg/kg body weight) and returned to incubation at 37 °C. The mortality was recorded daily for 5 days. If three or more larvae survived, the lowest dose was re-tested and the lowest antibiotic dose was assigned as therapeutic. If three or more larvae died, the antibiotic was tested against the same infective dose of bacteria at a higher dose (25 mg/kg body weight). The experiment was continued until a therapeutic dose was assigned or an antibiotic was ineffective against the infection. The values obtained were compared to values recommended for human use [14]: ampicillin—50–200 mg/kg body weight/day, ciprofloxacin—10–15 mg/kg body weight/day, tetracycline—25–50 mg/kg body weight/day and rifampicin—10–20 mg/kg body weight/day. Each step in the procedure included three control groups: untreated control, traumatized control (cuticle was pierced with a needle) and buffer-injected control.

### Toxicity testing procedure

**Fig. 2:**
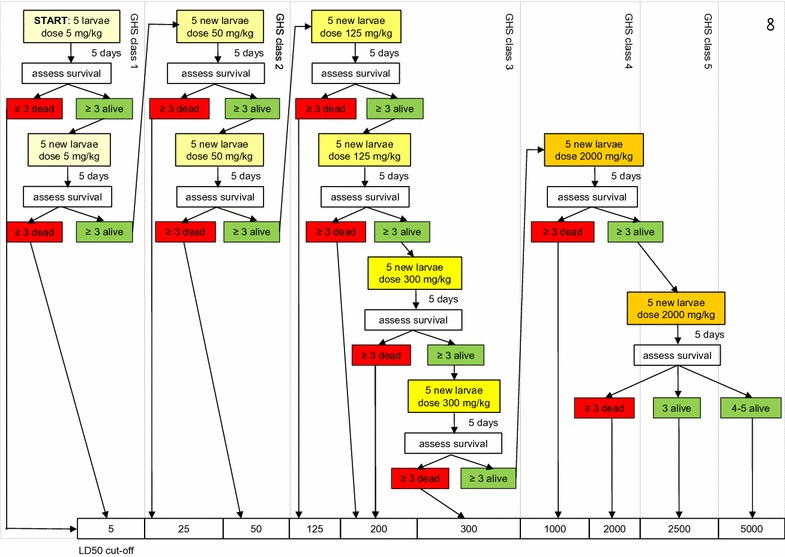
A flowchart representing consecutive steps in the acute toxicity test. A starting dose of 5 mg/kg body weight was administered and the insects were scored for mortality. If the mortality was over 40%, the compound was assigned the highest toxicity class. If the mortality is below 40%, the dose was re-tested and the testing continued until a toxic dose was established

A flowchart, adapted from the OECD guidelines for acute toxicity [15], was used to select the toxic dose of test compounds (Fig. 2). The acute toxicity testing was started by injecting five larvae with the initial dose of a compound (5 mg/kg body weight). Larval mortality was recorded daily. If three or more larvae died, the compound was assigned the highest toxicity class (GHS 1). If three or more larvae survived for 5 days, the toxicity testing was continued by re-testing the initial dose (5 mg/kg body weight) on a new cohort of larvae. If three or more larvae of the second cohort survived, a higher dose (25 mg/kg body weight) was tested in five fresh larvae. The experiment was continued until a toxic dose was established. If a compound was not toxic at the highest dose tested (2000 mg/kg body weight), the compound was classified as non-toxic. The obtained toxic dose was compared to a dose reported in Material Safety Data Sheet (MSDS) for the compound. Where possible the reported value used for the comparison was from mouse or rat via an intraperitoneal injection, when such data was absent the data from oral toxicity tests in a mammalian system was used. Each step in the procedure included three control groups: untreated control, traumatized control (cuticle was pierced with a needle) and buffer-injected control.

## Results and discussion

### Study design

Both the antibiotic efficacy trials and the toxicity testing were based on OECD guidelines for toxicity testing in mice and rats [15]. The guidelines have been adapted for the use with *G. mellonella* because they are a statistically robust method and allow for a streamlined workflow with a clear start and end point.

### Antibiotic efficacy

Initially the appropriate infective dose of bacteria was determined for each bacterial species. An infective dose was one that caused immune response observed as darkening of the cuticle and 60–80% lethality within 48 h, but not 100% lethality within 24 h. This was determined to be 5 × 10^6^ cfu in 10 µl for *E. coli*, *M. smegmatis* and *S. aureus*, and 5 × 10^4^ cfu in 10 µl for *P. aeruginosa*.

The efficacy of four antibiotics: ampicillin, ciprofloxacin, tetracycline and rifampicin, in *G. mellonella* larvae was assessed against four bacterial pathogens: Gram-positive *M. smegmatis* and *S. aureus*, and Gram-negative *E. coli* and *P. aeruginosa*. For each bacterial strain there was at least one antibiotic that is indicated in the treatment of an infection caused by that strain, and at least one that is predicted not to clear the infection to confirm that the antibiotic action, not the insect immunity, is responsible for the recovery.

Antibiotic efficacy testing was carried out as described in “Methods” section. The therapeutic dose of an antibiotic was determined when the antibiotic rescued the mortality caused by the bacterial infection. When even the highest dose of the antibiotic did not clear the infection the antibiotic was regarded as ineffective against those bacteria. The results are summarised in Table 2. There are four possible outcomes of the antibiotic testing: “(1) predicted dose of an antibiotic clears a bacterial infection, (2) a dose different from the predicted dose clears an infection, (3) antibiotic predicted to be ineffective is ineffective, (4) antibiotic predicted to be ineffective clears the bacterial infection. In most cases the antibiotics performed approximately as predicted, either treating an infection within the dose predicted or being ineffective against a resistant strain.

**Table 2:** Predicted and determined antibiotic susceptibility

Bacteria	Therapeutic dose (mg/kg body weight)							
	Ampicillin		Ciprofloxacin		Tetracycline		Rifampicin	
	Predicted	Test result	Predicted	Test result	Predicted	Test result	Predicted	Test result
*Escherichia coli*	50–200	Resistant	10–15	25	25–50	25	Resistant	Resistant
*Mycobacterium smegmatis*	Resistant	Resistant	Resistant	Resistant	Resistant	Resistant	10–20	Resistant
*Pseudomonas aeruginosa*	Resistant	Resistant	10–15	Resistant	Resistant	Resistant	Resistant	Resistant
*Staphylococcus aureus*	50–200	200	10–15	200	Resistant	50	10–20	25

It was found that ciprofloxacin, tetracycline and rifampicin performed as expected against *E. coli* infections. Both ciprofloxacin and tetracycline worked within the ranges prescribed for humans (10–15 and 25–50 mg/kg body weight/day respectively). Rifampicin was, as predicted, not effective as Enterobacteriaceae are intrinsically resistant to rifampicin. Ampicillin was expected to clear the infection at 50–200 mg/kg body weight, but it failed to treat the infection in Galleria. *P. aeruginosa* was resistant to all antibiotics used. *P. aeruginosa* is intrinsically resistant to ampicillin, tetracycline and rifampicin, and these antibiotics were tested as a negative control. The strain was expected to be sensitive to ciprofloxacin, which is active against Gram-negative pathogens, but when tested in the wax moth larvae *P. aeruginosa* was resistant to this drug. *M. smegmatis* was also resistant to all antibiotics used. Mycobacteria are intrinsically resistant to a range of antibiotics, and ampicillin, ciprofloxacin, and tetracycline were predicted to produce a resistant phenotype. Surprisingly rifampicin did not work, even though it is a standard treatment against mycobacterial infections. All antibiotics used were effective against *S. aureus*. Ampicillin worked at the high end of the spectrum normally prescribed for human use: 50–200 mg/kg body weight/day. Ciprofloxacin only cleared the infection at a concentration nearly 20 times higher than the dose recommended for human use. Tetracycline was predicted to be ineffective but it cleared the *S. aureus* infection at 50 mg/kg/body weight. There are a number of potential explanations for this, e.g. tetracycline susceptibility could have been caused by the loss of a tetracycline-resistance determinant from the *S. aureus* strain tested. Rifampicin, which is a standard treatment for methicillin-resistant *S. aureus* (MRSA), was effective at a low concentration.

These antibiotic efficacy studies were based on existing literature [2]. *G. mellonella* larvae have been previously used to study bacterial virulence and susceptibility to antibiotics and our study confirmed that the larvae are a suitable host for antibiotic efficacy studies. Additionally, we determined that the antibiotic therapeutic dose established in *G. mellonella* often matches the doses recommended for use in humans.

Our experiments support the proposal that antibiotic efficacy testing can be done in *G. mellonella* and the therapeutic doses recommended for human use can be translated to doses in the wax moth larvae. In most cases the exact dose recommended for clinical treatment of a systemic infection can be calculated for larval body weight and can clear an infection. Such correlation is possible because often the mechanisms of microbial virulence are not host-specific. Where the predictions did not match the outcomes, there are a variety of potential explanations, e.g. differences in immune responses in different organisms, etc. Previous studies have shown that the fungal pathogen *Candida albicans* uses the same repertoire of effectors, involved in fungal virulence and yeast-to-hypha transition, against insects and mammals [4]. Similarly, the bacterial pathogen *P. aeruginosa* employs a similar set of virulence genes to overcome the immune system of wax moth larvae and mice [6] and the larvae can be used to identified virulence factors required for an infection in mammals.

### Toxicity testing

11 compounds were provided by Inspiralis Ltd. to test for their toxicity in *G. mellonella* larvae. The compounds were initially tested blind, i.e. they were supplied in numbered tubes and only after the test procedure was completed were the numbers linked to compound names. The aim of this approach was to avoid bias, i.e. assigning lower toxic doses to known toxic compounds and higher ones to safer compounds.

Insects (5–10 larvae) were injected with 10 µl compound into the hindmost proleg. Intraperitoneal injection was used in the procedures to strictly control the amount of toxic compound or bacterial pathogen that the larvae were exposed to. Alternative approaches, not applied in this study, use feeding procedures [16] or contact exposure [17]. The quantification of exposure to a compound is less precise for such procedures, but it is sometimes a more appropriate method when a route of exposure is known. For example, pesticide toxicity in insects testing normally employs feeding studies [18] as it is the normal route of exposure.

Insects were injected with low doses (5 mg/kg body weight) of the compounds first and the mortality was recorded daily for 5 days. If no mortality was observed new groups of larvae were injected with compounds at 50 mg/kg body weight and the mortality was recorded daily for 5 days again. When mortality was observed in 60% or more of the larvae, the compound was re-tested at that same concentration to confirm the toxicity. When mortality below 60% was observed, the compounds were tested subsequently at 125, 300 and 2000 mg/kg body weight. Each compound was assigned an LD_50_ value (Table 3) and the values were compared to toxicity data available in the MSDS pages. No compounds were tested above 2000 mg/kg body weight in line with OECD guidelines [15]. Such high compound concentrations introduce solubility issues and are discouraged in the guidelines as unnecessary and unethical. Generally, compounds with no indication of toxicity at 2000 mg/kg body weight are considered non-toxic.

**Table 3:** Toxicity of compounds used in the trial

Compound	LD_50_ (mg/kg body weight)^a^				
	*G. mellonella*	Rat		Mouse	
	Intraperitoneal	Oral	Intraperitoneal	Oral	Intraperitoneal
Amsacrine	40	100		243	
Chloroquine	125^b^	623		500	
Ciprofloxacin	>2000	>2000		>2000	
DMSO	100	14,500		7920	
Doxorubicin	5.5		16	698	1.2
Etoposide	100	1784	58	215	
Glucose	>2000	25,800			
Novobiocin	100	3500		962	
Sodium chloride	>2000	3000		4000	
Streptomycin	300	430		430	
Tetracycline	>2000	6443	318	2759	368

^a^LD_50_ values determined in the test were compared to values available in Material Safety Data sheets provided with the compounds. Blank space indicates the data are not available

^**b**^Compounds tested at >100 mg/Kg were initially tested in 50% DMSO (which is toxic to *G. mellonella*) and then re-tested at lower [DMSO]s such that the final [DMSO] was 10%

### Comparison of toxicity testing in *G. mellonella* with studies in rodents

There are three possible outcomes of the comparison of toxicity in the wax moth larvae and in rodents. Firstly, the toxicity can be the same or very similar. Secondly the toxic doses in *G. mellonella* can be higher than the ones in rodents, and finally the toxic doses in *G. mellonella* can be lower than the doses for rodents. Our standard injection medium, 50% DMSO in water, was lethal to wax moths at a dose equivalent to 100 mg/kg body weight (a 4 μl injection of a stock solution or more than 2 μl of pure DMSO per injection). All compounds toxic above 100 mg/kg body weight had to be re-tested in a modified injection medium with a decreased amount of DMSO (<10% final; Table 3).

For most compounds the toxicity determined in this experiment correlates well with toxicity reported in the MSDS pages available for each of the compounds. The compounds with the lowest LD_50_s in mammals were the most toxic for the wax moth larvae (Table 3). The most toxic compounds had the lowest LD_50_ values: the value for doxorubicin fell within the range established by experiments in mammals (5.5 mg/kg in Galleria vs 1.2 and 16 mg/kg in mice and rats respectively) and for amsacrine the value was lower (40 mg/kg via intraperitoneal injection vs 100 mg/kg in rats and 243 mg/kg in mice via oral exposure). Similar correlations between LD_50_ values established in *G. mellonella* and LD_50_ values from MSDS pages was also observed for etoposide, ciprofloxacin, and streptomycin.

Glucose, sodium chloride and tetracycline were not toxic to *G. mellonella* at 2000 mg/kg body weight, and in line with the guidelines for toxicity testing in murine models, the compounds were not tested above this concentration. The LD_50_ values established in rats and mice were higher than 2000 mg/kg body weight. The values obtained for novobiocin and chloroquine were thirty to ten times smaller than the mammalian LD_50_, but the only data available is from oral exposure, making the values difficult to compare. The same is true for 50% DMSO, which is more toxic to *G. mellonella* larvae than to rats and mice.

An important limitation of using *G. mellonella* larvae in toxicity testing, which also applies to all other toxicity testing systems, is that the toxicity cannot be directly tested in humans and it is not known how experimental LD_50_ values correlate to human values or even if the mechanisms of toxicity are the same. There are numerous mechanisms of systemic toxicity and they are poorly understood. In some cases, the cause of toxicity is alike in different systems. For example, doxorubicin is a DNA intercalator and poisons different organisms at low doses.

The use of DMSO as an injection medium in the procedure is another limitation to the study. 50% DMSO was lethal to wax moths above 100 mg/kg body weight when used as an injection medium. The test cannot correctly assign LD_50_ to mildly toxic compounds in 50% DMSO (for example antibiotics ciprofloxacin and chloramphenicol) and non-toxic compounds (glutamic acid, glucose, sodium chloride) because of the side effects of the DMSO injection. Decreasing the amount of DMSO per injection (to 10% or less), maintains the solubility of the compounds tested without compromising the test procedure. Alternatively, the solvent effects can be subtracted from the compound effects using statistical methods. Restricting background mortality (mortality in untreated control groups) not only lowers the experimental noise but also aligns better with the guidelines on the use of laboratory animals.

Overall our experiments have shown that Galleria larvae can be used in acute toxicity testing, providing data more cheaply and quickly than traditional testing systems. Testing in *G. mellonella* is unlikely to fully replace toxicity testing in mammals, but it is a convenient step between in vitro tests and testing in mammals, adding more complexity to the former and statistical robustness to the latter.

## Conclusion

In summary, our experiments support that proposal that antibiotic efficacy can be tested in vivo in *Galleria mellonella* larvae. We established that the doses recommended for use in humans can be effective in systemic infections in the larvae and that the acute toxicity of compounds in wax moth larvae correlates to the toxicity in mice and rats. *G. mellonella* is an organism that can be easily adopted in various tests. It cannot fully replace mammalian models, but it is much cheaper and can provide the statistical robustness current animal models lack.

## Abbreviations

DMSO: dimethyl sulfoxide
MSDS: material safety data sheet
PBS: phosphate-buffered saline
OECD: Organisation for Economic Co-operation and Development

## References

[1] Graham ML, Prescott MJ (2015). The multifactorial role of the 3Rs in shifting the harm-benefit analysis in animal models of disease. Eur J Pharmacol.

[2] Cook SM, McArthur JD (2013). Developing *Galleria mellonella* as a model host for human pathogens. Virulence.

[3] Thomas RJ, Hamblin KA, Armstrong SJ, Muller CM, Bokori-Brown M, Goldman S, Atkins HS, Titball RW (2013). *Galleria mellonella* as a model system to test the pharmacokinetics and efficacy of antibiotics against *Burkholderia pseudomallei*. Int J Antimicrob Agents.

[4] Brennan M, Thomas DY, Whiteway M, Kavanagh K (2002). Correlation between virulence of *Candida albicans* mutants in mice and *Galleria mellonella* larvae. FEMS Immunol Med Microbiol.

[5] Fuchs BB, O’Brien E, Khoury JB, Mylonakis E (2010). Methods for using *Galleria mellonella* as a model host to study fungal pathogenesis. Virulence.

[6] Jander G, Rahme LG, Ausubel FM (2000). Positive correlation between virulence of *Pseudomonas aeruginosa* mutants in mice and insects. J Bacteriol.

[7] Cytrynska M, Zdybicka-Barabas A, Jakubowicz T (2007). The involvement of protein kinase A in the immune response of *Galleria mellonella* larvae to bacteria. Acta Biochim Pol.

[8] Browne N, Heelan M, Kavanagh K (2013). An analysis of the structural and functional similarities of insect hemocytes and mammalian phagocytes. Virulence.

[9] Peleg AY, Jara S, Monga D, Eliopoulos GM, Moellering RC, Mylonakis E (2009). *Galleria mellonella* as a model system to study *Acinetobacter baumannii* pathogenesis and therapeutics. Antimicrob Agents Chemother.

[10] Harding CR, Schroeder GN, Collins JW, Frankel G (2013). Use of *Galleria mellonella* as a model organism to study *Legionella pneumophila* infection. J Vis Exp JoVE.

[11] Megaw J, Thompson TP, Lafferty RA, Gilmore BF (2015). *Galleria mellonella* as a novel in vivo model for assessment of the toxicity of 1-alkyl-3-methylimidazolium chloride ionic liquids. Chemosphere.

[12] Desbois AP, Coote PJ (2011). Wax moth larva (*Galleria mellonella*): an in vivo model for assessing the efficacy of antistaphylococcal agents. J Antimicrob Chemother.

[13] Hill L, Veli N, Coote PJ (2014). Evaluation of *Galleria mellonella* larvae for measuring the efficacy and pharmacokinetics of antibiotic therapies against *Pseudomonas aeruginosa* infection. Int J Antimicrob Agents.

[14] Szczeklik A, Gajewski P. Interna Szczeklik 2014. Textbook of internal medicine in 2014. 2014.

[15] OECD. Guidance document on acute oral toxicity testing. OECD Publishing; 2001.

[16] OECD. Test No. 237: Honey bee (*Apis mellifera*) larval toxicity test, single exposure. OECD Publishing; 2013.

[17] OECD. Test No. 214: Honeybees, acute contact toxicity test. OECD Publishing; 1998.

[18] Charbonneau C, Cote R, Charpentier G (2007). Effects of azadirachtin and of simpler epoxy-alcohols on survival and behaviour of *Galleria mellonella* (Lepidoptera). J Appl Entomol.

